# Use of H-Index and Other Bibliometric Indicators to Evaluate Research Productivity Outcome on Swine Diseases

**DOI:** 10.1371/journal.pone.0149690

**Published:** 2016-03-01

**Authors:** Ivan Díaz, Martí Cortey, Àlex Olvera, Joaquim Segalés

**Affiliations:** 1 IRTA, Centre de Recerca en Sanitat Animal (CReSA, IRTA-UAB), Campus de la Universitat Autònoma de Barcelona, 08193 Bellaterra, Barcelona, Spain; 2 Institut de Recerca de la Sida, IrsiCaixa—HIVACAT, Carretera del Canyet, s/n, 08916 Badalona, Spain; 3 UAB, Centre de Recerca en Sanitat Animal (CReSA, IRTA-UAB), Campus de la Universitat Autònoma de Barcelona, 08193 Bellaterra, Barcelona, Spain; 4 Departament de Sanitat i d’Anatomia Animals, Universitat Autònoma de Barcelona, 08193 Bellaterra (Cerdanyola del Vallès), Spain; Institut National de la Recherche Agronomique, FRANCE

## Abstract

H-index is the most commonly applied tool to evaluate scientific productivity. In this study, the use of the H-index to evaluate scientific production in swine veterinary medicine was explored. A database of 137 pig infectious agents was constructed, including its taxonomic division, zoonotic potential, status as emerging pathogen and whether it was OIE-listed. The H-index and the total number of citations were calculated for those pathogens, the location of the affiliation of the first author of each paper included in the H-index core was registered and, for the ten pathogens with the highest H-index, evolution over time was measured. H-index values were compared to the M quotient, A-index, G-index, HG-index and the G/H ratio. H-indices were found to be severely affected by search accuracy and the database was hand curated. Swine pathogen H-indexes were highly dispersed ranging from 0 to 106 and were generally higher for pathogens causing endemic diseases in large pig producing countries. Indeed, the three top pathogens were *Escherichia coli*, *Porcine reproductive and respiratory syndrome virus* and *Porcine circovirus type 2* with H-indices 106, 95 and 85, respectively. H-indices of viruses and bacteria were significantly higher (*P*<0.001) than other pathogen types. Also, non-zoonotic pathogens had higher H-indices than zoonotic pathogens (p<0.009) while no differences could be found for being listed by the OIE. For emerging diseases, only non-emerging viruses had higher H-index (p = 0.02). The study of H-indexes over time revealed three general patterns and that they had increased mainly after the 1980’s. As expected, there were strong geographic patterns in terms of authorship and North America (38%) and Europe (46%) coped the majority of the papers. Finally, in order to quantify the contribution of a subject to a specific field, a new index “*Deciphering Citations Organized by Subject”* (Dcos) is proposed.

## Introduction

The measure of the scientific quality and productivity of a particular researcher or research team has been traditionally a controversial issue. The topic is not trivial as it can be used by managers to evaluate professional promotions within a research institution, or by funding agencies and scientific panels to prioritize projects, grants and fellowships. The simplest way to tackle this challenge is considering the number of publications, the overall and mean number of citations for those publications, or the impact factor of the journal. However, these classical forms have many drawbacks [1–3]. Additionally, numerous bibliometric indices have been proposed, being the H index the first to be created and the most popular [4]. Based on a set of publications and their number of citations, the H index is defined as the number of published papers (N) that have been cited N or more times. Therefore, this value combines both the quantity (the number of papers) and the quality (the number of citations) of the publications. As soon as it was proposed, H index was widely considered as a factual and accurate parameter to quantify an individual’s scientific output [1,3,5–8]. Initially proposed for individual researchers, H index can be applied to any set of papers [9,10]: journals [11], countries [12], and even within a specific field, such as compounds in physics [13]. More recently, some authors have proposed H-index as a quantitative indicator to evaluate the impact of pathogens and infectious diseases or, at least, its relative scientific interest [14–16]. In those articles, authors demonstrated a significant correlation between the H-index and other indicators or projects that measure the impact of pathogens in humans and in animals, such as *Disability Adjusted Life Year (DALY)* -an indicator developed by the World Health Organization that measures one year of health life lost and deaths from disease [15] -, *Health Adjusted Life Year (HALY)*, which lists three measures of disease burden for infectious diseases that have occurred in Ontario [14,17], and DISCONTOOLS -project funded by the European Commission that investigates the impact of more than 50 domestic animals diseases with the aim to be a tool to prioritize research [16] -. A general conclusion was that H-index is a useful tool if complemented with qualitative criteria for ranking human and animal pathogens.

The advantages of the H-index to measure the impact of pathogens were evident: it is simple, comprehensible and quick to calculate, and it is a robust cumulative indicator, since increasing citations in the top-cited articles or publications without citations do not have a direct effect on the H-index score. Furthermore, some disadvantages of the H-index when used to evaluate researchers do occur to a lesser degree when applied to pathogens. For instance, a certain pathogen cannot have a high H-index and increase without a steady increase in publications [14]. Even though, H-index still has several inconveniences when used for evaluating the impact of pathogens mostly intrinsically related to the H-index definition and concept. On one hand, H-index places emerging pathogens in a disadvantageous situation, since some time is needed to accumulate citations. On the other hand, some pathogens defined long time ago may have reached a high H-index score in the past and rest on its laurels since then. Additionally, the publication dynamics of the papers is not considered. Thus, several indicators other than H-index have been proposed to overcome most of these limitations [3,9,18], such as M quotient [4], A-index [19] and G-index [20], among others. However, these alternatives have never been measured to evaluate pathogen impact [14].

In swine, several parameters can be used to evaluate the pathogen impact, like disease cost on the productivity, treatment or control/eradication, prevalence, mortality, zoonotic potential, severity and global or regional spread, among others. Some of these features and figures can be extremely time-consuming, hardly computable, and quite often they are not available. If a large pathogen dataset needs to be evaluated, the resulting scenario can be particularly hard and complicated. Whether considered as a single or a complimentary evaluation figure, and especially when the formerly cited parameters cannot be calculated or are missing, H-index score could be a very useful tool to compare the interest of the numerous pathogens affecting swine. In the present study, the importance and impact of porcine pathogens using the H-index were calculated, compared and analysed. Swine infectious agents were characterized according to its taxonomic, emerging, zoonotic and OIE (World Organization for Animal Health) communication status, and main H-index scores within groups were compared. The temporal evolution and geographical distribution of the papers contained in these H-indices cores were also examined. As far as the authors’ know, the pathogen impact was for first time also measured using indicators other than H-index (M quotient, A-index, G-index, HG-index and G/H ratio). Moreover, considering that none of these indices reflects the particular contribution within the H-index from an author, an institute or a country, a new index “*Deciphering Citations Organized by Subject*” (Dcos) is proposed. Overall, the results obtained in the present study can be useful to know the hottest topics in swine research. In addition, Dcos scores can be used to evaluate if an author, an institute or a country have a significant impact in a particular research pathogen or topic.

## Materials and Methods

### Selection and grouping of pathogens

The present study comprises all known organisms that cause infection/infestation in pigs (from now on, infectious agent or pathogen). The major internationally-recognized reference book in swine veterinary medicine “Disease of swine” [21] was mainly used to construct the final database of pig infectious agents. This database included the pathogen name, its taxonomic division, the information on whether it had zoonotic potential, if it was emerging or not emerging, and whether it caused an OIE-listed disease or not. Pathogens were defined as non-emerging or emerging if they have been firstly described in pigs before or after the last 20 years, respectively [15] and the grouping into OIE-listed diseases was done according to the information available at the OIE website [22]. The list and grouping were completed using institutional websites from two European projects included in the 7th Framework Programme: the ENHanCEd Infectious Diseases (EID2) [23] and DISCONTOOLS [24]. EID2 is an evidence-based database that uses publicly available databases to compile data on vectors, hosts and their pathogens. Among other information, it collects data on spatial and temporal distributions of infectious agents and bibliographic and other supporting evidences. The impact of the spatial distribution of pathogens on H-index scores was evaluated by collecting its distribution (worldwide, continental or national) from EID2. The DISCONTOOLS project (Development of the most effective tools to control infectious diseases in animals) aims to be a tool to target research funding and prioritize research. Both EID2 and DISCONTOOLS sources were already used in previous papers analyzing human and domestic animal pathogens in Europe [16], and emerging hazards in North-America [14], according to the H-index scores. Also, other institutional and governmental websites from the Netherlands [25], Australia [26], and the USA [27] and the internationally-recognized reference publication in veterinary medicine The Merck Veterinary Manual [28] as well as Meng [29], were used to complete the list and the grouping of pathogens.

Using the abovementioned sources, the H-index was calculated for a total of 137 swine pathogens: *Viruses* (n = 52), *Bacteria* (n = 39), and *Other* (n = 46), in which helminthes, protozoa, external parasites (classes Insecta and Arachnida), and fungi were included. Overall, most of them were considered non-emerging (86.1%) and/or zoonotic (62.8%), as summarized in Table 1. In particular, *Virus* was an exception as majority of the members included in this group were assigned in the Non-zoonotic status (65.8%). Regarding Emerging group (n = 19; 13.9%), it was mainly composed by viruses (18 *Virus*, 1 *Bacteria*, 0 *Other*). Finally, only 24 out of 137 (17.5%) of the pathogens incorporated in the study were included in the OIE-listed diseases (2015), most of them viruses.

**Table 1:** Distribution of pathogens. Taxonomic divisions (number and percentage) of infectious agents used in the study and whether they cause an emerging (less than 20 years from first description in pig) or non-emerging associated diseases, with zoonotic potential or as OIE-listed disease (2015) according to sources described in “Selection and grouping of pathogens”.

	TOTAL		Emerging	Not emerging	Zoonotic	Not zoonotic	OIE listed	Not OIE listed
	Number	Percentage						
**Virus**	52	38.0%	18 (34.6%)	34 (65.4%)	18 (34.6%)	34 (65.4%)	13 (25.0%)	39 (75.0%)
**Bacteria**	39	28.4%	1 (2.6%)	38 (97.4%)	28 (71.8%)	11 (28.2%)	5 (12.8%)	34 (87.2%)
**Other**^1^	46	33.6%	0 (0%)	46 (100%)	40 (87.0%)	6 (13.0%)	6 (13.0%)	40 (87.0%)
**TOTAL**	137	100%	19 (13.9%)	118 (86.1%)	86 (62.8%)	51 (37.2%)	24 (17.5%)	113 (82.5%)

^**1**^ Helminthes, protozoa, external parasites and fungi

### Calculation of the H-index score of pathogens

The bibliographic software package Institute Scientific information’s Web of Science (WOS) [30] was used to calculate H-index scores, since previous works demonstrated that other bibliographic databases as SCOPUS and Google Scholar [16] or PubMed [14] yielded not identical but highly correlated results. All searches, which were undertaken in March 2015, were restricted to articles published in English between years 1900 and 2015, both inclusive.

Due to the existence of multiple names, synonymous and acronyms used for a given infectious agent, or even the use of compilation of lesions to describe the disease caused by a pathogen as a synonymous of the name of the pathogen itself, searches were done using the following terms specified in quotation marks (“”): pathogen complete name, alternative names, acronym/s, common names, their synonymous, and the associated disease/s, not only according to NCBI Taxonomy [31] but also using other sources such as websites described in “Selection and grouping of pathogens”. Particularly for viruses, the International Committee on Taxonomy of Viruses website [32] was also used. Terms “porcine”, “pig” or “swine” where used for each pathogen to delimit the search to the papers related to pigs. The Boolean options “AND” and “OR”, and “NOT” for exclusion terms, were used when necessary to link multiple search terms.

As an example, the following phrases were used for one of the most worldwide costly disease in swine industry [33]:“*Porcine reproductive and respiratory syndrome virus*” OR “PRRS” AND “virus” OR “PRRSV” OR “Mystery disease of swine” OR “MDS” OR “Lelystad virus” OR “*porcine arterivirus”* OR “Swine infertility and respiratory syndrome” OR “SIRS”. Thus, changes in terminology since the virus was firstly described were accounted. In the example, some of these phrases resulted in publications not related to the PRRS virus, i.e. PRRs-pattern recognition receptors. Also, using other phrases or searching for other pathogens, some listed publications could not be related to the given pathogen or to pigs. To correct this bias, papers in the automatically generated lists were curated one by one to ensure database accuracy. Publications related to pork (pig meat), feral swine or fungi contaminating pig’s feed were not considered.

Apart from H-index scores, the total number of citations as well as mean citations per paper included in each H-index core were directly obtained from the WOS output. The quartiles of the journals where papers were published were also investigated [34].

### Research productivity by continents and countries

National and continental contribution to the research productivity on swine pathogens was evaluated by determining the location of the first author affiliation of all papers included in each H-index using WOS and PubMed [35]. Three-letter country codes defined and published by the International Organization for Standardization (ISO-3166-1) [36] were used to represent each country.

### Changes in H-index scores over time

H-indices over time were measured for the ten pathogens with the highest H-index. For this purpose, data generated by WOS was used to calculate H-index scores and the percentage of changes in the index year after year, from the first paper published for each pathogen to 2015 inclusive.

### Comparison of H-index with other indices

In order to compare the ranking generated by the H-index, scores with prioritization of pathogens obtained using other indicators—M-quotient, A-index, G-index, HG-index as well as G/H ratio—were calculated for the ten pathogens with the highest H-indices.

To avoid the conjecture that the H-index can be directly proportional to career length, Hirsch [4] proposed the M quotient to “compare scientists with different lengths of scientific careers”. This indicator, which is calculated dividing the H-index by number of years of research activity, should also be applied evaluating research productivity outcome in pathogens, since there were noticeable differences concerning year of their first description. For that, H-index of a given pathogen was divided by years from publication of the oldest paper included in its H-index to calculate M quotient.

A-index, defined as the mean number of citations of papers that are included in the H index [19], was directly and automatically generated by WOS from the list of publications included in the H-index. Regarding G-index, a new longer list of publications for each pathogen was needed; this new list was obtained applying same terms and parameters described for H-index. New list was needed since, if a set of papers were ranked in decreasing order of the number of citations that they received, the G-index is defined as “the largest number such that the top G articles received (together) at least G^2^ citations” [37,38]. Therefore, list of publications for G-index calculation is always longer than for H-index.

Using H- and G-index scores, the HG-index (HGi = sr(HxG); [20]) was calculated, which avoids the big influence that a very successful paper can introduce in the G-index. The G/H ratio calculates the relative increase of G with respect to H [38].

### Statistical analyses

Descriptive statistics were done using the software package Microsoft® Office Excel® 2007. Statistical analyses and box-plot representations were done using the statistical software package Statgraphics v.17.1.08 (Statpoint Technologies, http://www.statgraphics.com). The normality of every dataset was estimated with the Shapiro-Wilk and the Kolmogorov–Smirnov tests. The comparisons of H-index means for every factor considered (taxonomy, emergence, zoonotic potential and OIE-list) were carried out with Mann–Whitney U test when 2 groups were compared or the Kruskal–Wallis test when comparisons were made among 3 groups. The limit of statistical significance was established by a *P-value*<0.05.

## Results

### Overall distribution of swine pathogens according to the H-index scores

**Fig 1:**
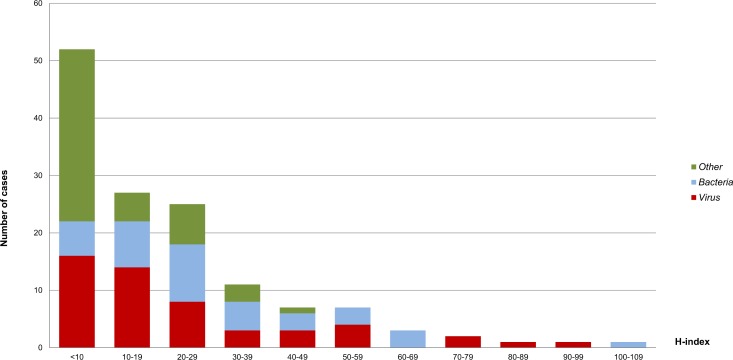
Frequency histogram. Frequency of the H-index scores for pig infectious agents according to taxonomic groups: *Virus*, *Bacteria* and *Other* (Helminthes, protozoa, external parasites and fungi).

The H-index scores of the swine pathogens ranged from 0 to 106 and were highly dispersed, with a global mean ± standard deviation (SD) of 20.96 ± 20.85 and a median of 14. Out of a total of 137 pathogens included in the study, 104 (76%) had an H-index lower than 30, and more than a half (n = 79; 57.7%) had an H-index lower than 20. Only 15 pathogens had H-indices higher than 50; seven bacteria and eight viruses. Just one pathogen (*E*. *coli)* had an index over 100 (Fig 1 and Table 2).

**Table 2:** Swine infectious agents with the top 20 H-index scores. Rank according to the H-index, infectious agent name, taxonomic group, H-index, total number of citations and mean number of citations of papers ± standard deviation (SD) for the publications included in the H-index core, and mean quartile of the journals ± SD where papers were published as well as origin (country) of the first author affiliation for the papers included in the H-indices for each infectious agent (top three countries and rest).

RANK	Infectious agent	Group	H-index	Total Citations	Mean Citations ± SD	Mean Quartile ± SD	COUNTRIES 1st, 2nd and 3rd	REST
**1**	***Escherichia coli***	BACTERIA	106	24023	153.0 ± 89.4	1.4 ± 0.9	USA (42.4%), GBR (18.9%), CAN (6.6%)	32.1%
**2**	***Porcine reproductive and respiratory syndrome virus* (PRRSV)**	VIRUS	95	16398	172.6 ± 114.6	1.6 ± 0.8	USA (53.7%), NLD (8.4%), CAN (7.4%)	30.5%
**3**	***Porcine circovirus type 2* (PCV2)**	VIRUS	85	14068	163.6 ± 84.4	1.8 ± 0.9	USA (31.8%), ESP (17.6%), GBR & CAN (14.1%)	22.4%
**4**	***Swine influenza virus* (SIV)**	VIRUS	79	16008	202.6 ± 295.6	1.3 ± 0.7	USA (60.7%), GBR (11.4%), CHN (8.9%)	19.0%
**5**	***Classical swine fever virus* (CSFV)**	VIRUS	72	8689	120.7 ± 66.9	1.3 ± 0.6	NLD (34.7%), DEU (26.4%), USA (8.3%)	30.6%
**6**	***Actinobacillus pleuropneumoniae***	BACTERIA	64	7740	120.9 ± 100.6	1.6 ± 1.0	CAN (32.8%), USA (23.4%), CHE (15.6%)	28.2%
**7**	***Streptococcus suis***	BACTERIA	62	10739	74.6 ± 83.4	1.6 ± 0.9	CAN (32.5%), NLD (24.2%), CHN (9.7%)	33.6%
**8**	***Salmonella* Typhimurium**	BACTERIA	61	8038	131.8 ± 105.6	1.4 ± 0.8	USA (41.0%), DNK (21.3%), NLD & GBR (8.2%)	21.3%
**9**	***Aujeszky's disease virus* (ADV)**	VIRUS	58	4498	86.2 ± 29.5	1.6 ± 0.9	NLD (25.9%), USA (19.0%), DEU (17.2%)	37.9%
**10**	***Foot-and-mouth disease virus* (FMDV)**	VIRUS	56	6702	119.7 ± 61.2	1.6 ± 0.8	GBR (41.0%), USA (25%), DNK & ESP (7.1%)	19.8%
**11**	***African swine fever virus* (ASFV)**	VIRUS	56	4802	85.7 ± 47.8	1.7 ± 0.9	ESP (46.4%), USA (25%), GBR (21.4%)	7.2%
**12**	***Pig endogenous retrovirus* (PERV)**	VIRUS	55	7578	137.8 ± 128.8	1.4 ± 0.7	USA (41.8%), DNK (29.1%), GBR (20%)	9.1%
**13**	***Mycoplasma hyopneumoniae***	BACTERIA	51	7392	62.6 ± 76.5	1.5 ± 0.8	USA (41.1%), GBR (17.6%), BEL (7.8%)	33.5%
**14**	***Staphylococcus aureus***	BACTERIA	51	6742	78.4 ± 58.5	1.2 ± 0.7	NLD (35.2%), DEU (15.7%), USA (11.8%)	37.3%
**15**	***Campylobacter jejuni***	BACTERIA	50	7423	65.7 ± 57.4	1.5 ± 0.7	USA (36%), GBR (22%), DNK (6%)	36.0%
**16**	***Porcine rotavirus* (PoRV)**	VIRUS	49	5049	103.0 ± 67.7	1.4 ± 0.6	USA (51.0%), GBR (16.3%), JPN (8.1%)	24.6%
**17**	***Hepatitis E virus* (HEV)**	VIRUS	49	6392	130.4 ± 105.5	1.7 ± 0.9	USA (36.7%), JPN & NLD (8.2%)	46.9%
**18**	***Enterococcus faecium and E*.*spp***	BACTERIA	47	6052	67.2 ± 59.2	1.2 ± 0.4	DNK (31.2%), USA & DEU (12.5%)	43.8%
**19**	***Pasteurella multocida***	BACTERIA	42	4251	48.9 ± 19.9	2.0 ± 1.2	USA (28.6%), GBR (19.0%), DNK (11.9%)	40.5%
**20**	***Porcine parvovirus Infection* (PPV)**	VIRUS	41	3218	78.5 ± 62.5	1.5 ± 0.6	USA (58.5%), ESP & GBR & CAN (7.3%)	19.6%
**22**	***Toxoplasma gondii***^1^	OTHER	40	4562	93.0 ± 164.1	1.7 ± 0.9	USA (52.7%), NLD (7.5%)	39.8%

^1^ From those included in group *Other*, pathogen with the highest H-index

Particularly, 11 out of 52 viruses (21.1%) had an H-index higher than 40, 25 (48.1%) between 10 and 39, and 16 (30.8%) had an index lower than 10. Regarding bacteria, 10 (25.6%) had an index higher than 40, 23 (59.0%) between 10 and 39, and only 6 (15.4%) had an index lower than 10. Finally, just one pathogen included in *Other* (*Toxoplasma gondii*) had an H-index of 40 (2.2%), 15 (32.6%) had an index between 10 and 39, whereas most of the pathogens in this group (n = 30; 65.2%) had an H-index lower than 10.

**Fig 2:**
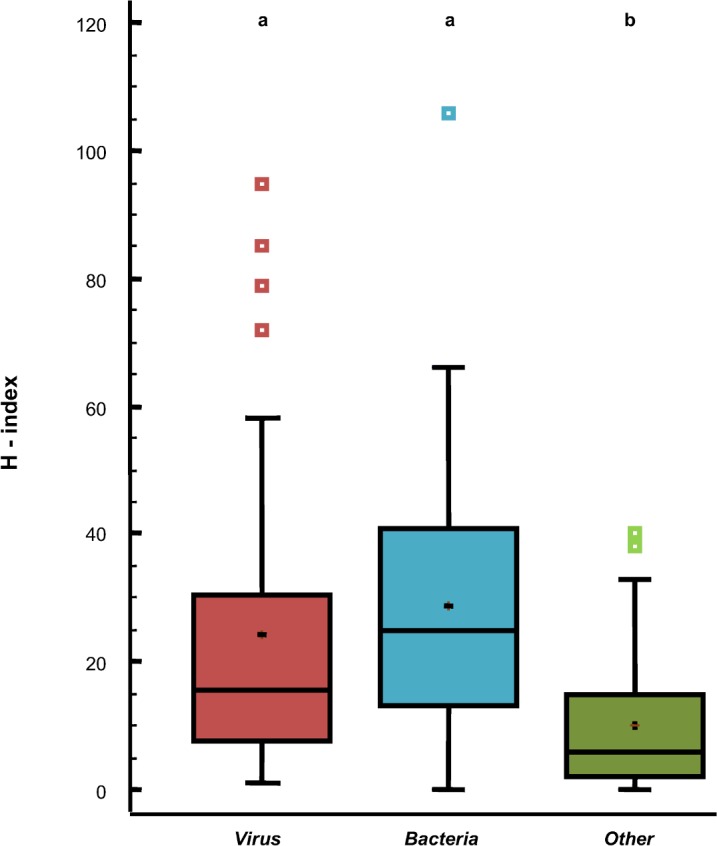
Box plot showing H-index score quartiles by taxonomic division. + Mean. a>b (*P*<0.001).

Grouping by taxonomy (Fig 2), H-indices for *Virus* and *Bacteria* (means ± sd = 24.2 ± 23.2 and 29.3 ± 21.2, respectively) were significantly higher than mean of H-index for *Other* (mean ± sd = 10.1 ± 10.8) (Kruskal-Wallis test, *P*<0.001). Median values for taxonomic groups were 15.5 for *Virus*, 26 for *Bacteria* and 6 for *Other*.

Concerning the other factors, there were no significant differences between groups when pathogens were distributed according to their emergence (emerging *vs* non-emerging: 16.2 ± 20.8 *vs* 21.5 ± 20.9, respectively) and OIE list (OIE-listed diseases *vs* non-OIE-listed diseases: 26.7 ± 24.7 *vs* 19.5 ± 19.9, respectively). In regards their zoonotic potential, mean of H-indices for non-zoonotic pathogens was significantly higher than that for zoonotic ones (25.1 ± 21.6 *vs* 18.3 ± 20.2, respectively; Mann–Whitney U test, *P*<0.009).

When organisms within a given taxonomy group were distributed according to their zoonotic potential and the OIE list, there were no significant differences (data not shown). In regards of their emerging status, there were significant differences only within viruses, where H-indices mean of non-emerging viruses was higher than that of emerging ones (28.0± 23.7 *vs* 17.0 ± 21.2, respectively; Mann–Whitney U test, *P* = 0.02).

### Top 20 ranking by H-index

Table 2 summarizes the top 20 swine pathogens ranked by their H-indices. None of the pathogens belonging to taxonomic groups included in *Other* were found in the top 20; the first one was *Toxoplasma gondii* found in rank 22 (H-index = 40), followed closely by *Taenia solium* (*Cysticercus cellulosae*) (rank 23; H-index = 38).

Considering all the infectious agents included in the study, 9 out of 86 (10.5%) classified as zoonotic, 2 out of 19 (10.5%) considered emerging, and 5 out of 24 OIE-listed (20.8%) were in the top 20. The remaining 4 agents in the top 20 were non-zoonotic, non-emerging and non-OIE-listed.

About 40% of the 52 diseases contained in the DISCONTOOLS database affect pigs and, therefore, they were included in the present study. Of these, 13 diseases were identified in the list of pathogens with the highest 20 H-indices. Within the ten pathogens with the highest H-index, eight were included in DISCONTOOLS.

The higher the H-index, the more papers sum for the total number of citations. Nevertheless, some pathogens with a low H-index had a high number of citations, since some of the papers within their H-index were highly cited. Thus, *E*. *coli* had the highest number of citations (24,023) but the mean of citations for the publications included in its H-index was the fourth (153.01 ± 89.45).

Regarding the mean quartile of the journals where papers included in the set of H-indices were published, it ranged from 1.2 ± 0.4 and 2.0 ± 1.2.

### Changes in H-index scores over time for the top ten pathogens

**Fig 3:**
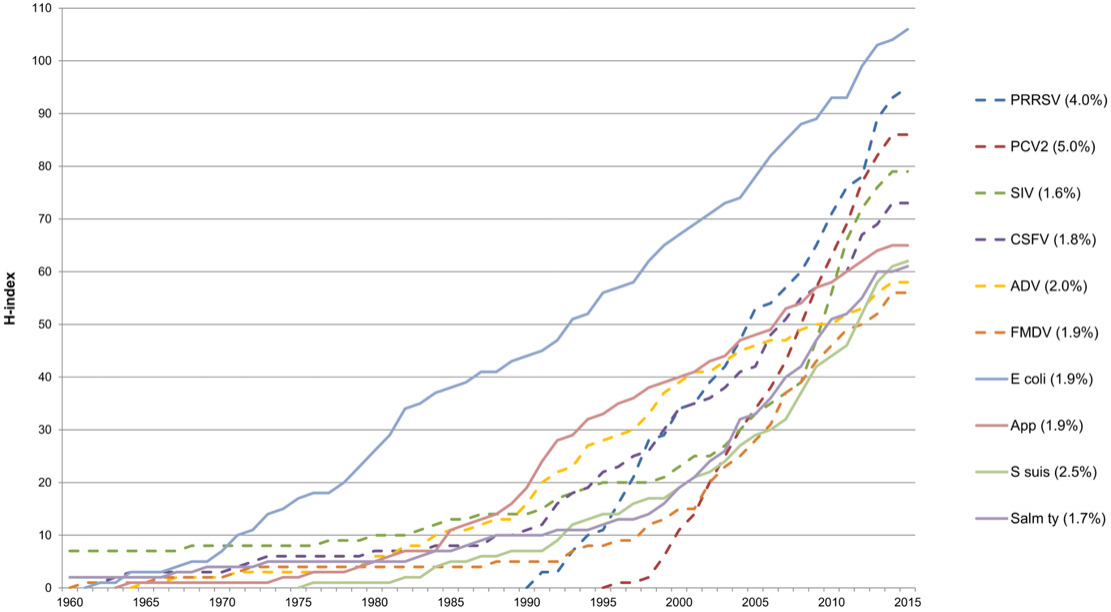
Evolution of H-index scores. Evolution of H-index scores by year from 1960 to March 2015 for the ten pig infectious agents with highest H-index, and mean of the percentage of increase by year from first paper published. PRRSV: *Porcine reproductive and respiratory syndrome virus*; PCV2: *Porcine circovirus type 2*; SIV: *Swine influenza virus*; CSFV: *Classical swine fever virus*; ADV: *Aujeszky’s disease virus*; FMDV: *Foot-and-Mouth disease virus*; E coli: *Escherichia coli;* App: *Actinobacillus pleuropneumoniae;* S suis; *Streptococcus suis;* Salm ty; *Salmonella* Typhimurium.

The evolution of H-index over time, as well as its average increase by year for the ten pathogens with the highest H-indices is shown in Fig 3. The H-index for these pathogens varied noticeably and three general patterns were defined. First, some pathogens that were initially described some decades ago demonstrated a slow and nearly plain evolution, even exhibiting a plateau shape during a variable time span, but experiencing abrupt and temporary increases -*Swine influenza virus* (SIV), *Foot-and-mouth disease virus* (FMDV), *Aujeszky’s disease virus* (ADV), *Classical swine fever virus* (CSFV), *Streptococcus suis*, *Salmonella* Typhimurium *and Actinobacillus pleuropneumoniae*-. Second, the H-index for *E*. *coli* is constant and gradual, but even in this case there were few abrupt increases (late 1970’s and early 1980s, and in the present decade). Third, for those pathogens that emerged more recently, such as *Porcine reproductive and respiratory syndrome virus* (PRRSV) or *Porcine circovirus type 2* (PCV2), their H-index scores increased steeply in few years, as can be seen in the percentage average annual H-index increase: 4.00% and 5.05%, respectively. Independently of these different shapes of evolution, and with the exception of *E*. *coli*, H-index scores for all pathogens were null or very low (<10) before 1980, but experienced an obvious increase during the 1980´s decade and afterwards.

### Research productivity by continents and countries

**Fig 4:**
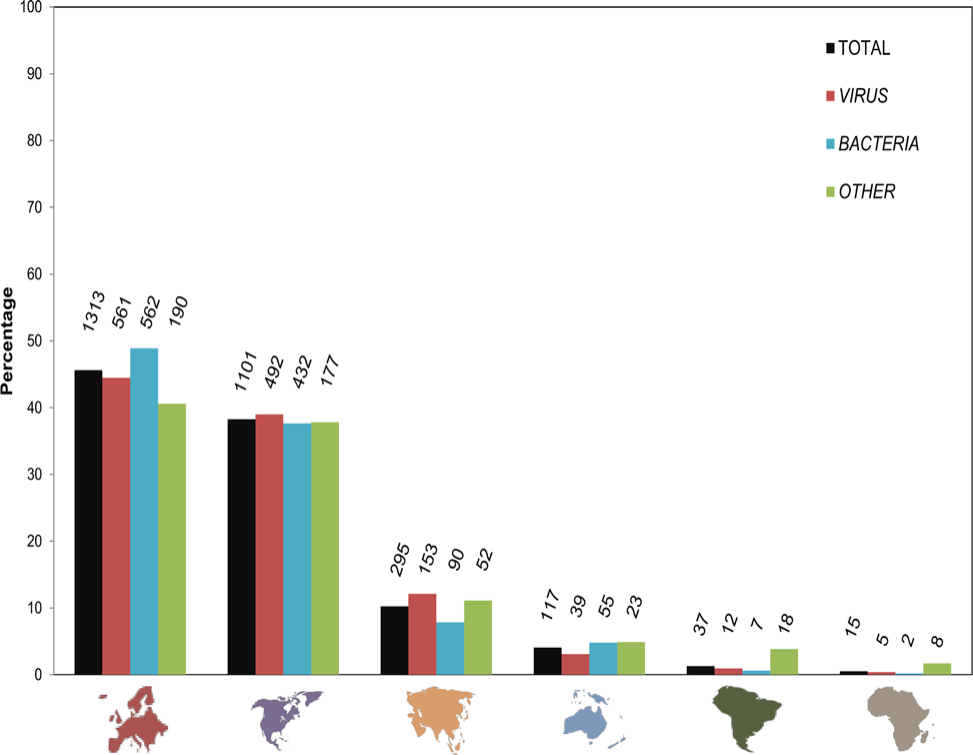
Distribution by continents (percentage) of papers included in the H-index scores. Bars show percentage of origin of the first author affiliation for the papers included in the H-indices; total papers (n = 2,878) (black), *Virus’* papers (n = 1,262) (red), *Bacteria’s* papers (n = 1,148) (blue) and O*ther*’s papers (n = 468) (green). Above each bar, absolute numbers of publications.

The contribution of a given continent and country to the research productivity on swine diseases was determined by the origin of the first author affiliation of each paper included in each the H-index (Fig 4 and Table 3, respectively). Contribution of countries for the pathogens with the 20 highest H-indices is also detailed in Table 2.

**Table 3:** Country (and continent) of origin of the first author affiliation (top ten). Country (and continent) of origin of the first author affiliation (top ten) for the 2,878 papers included in the H-indices searched for 137 infectious agents; 1,262 papers for the 52 organisms comprising Virus, 1,148 for 39 organisms in Bacteria, and 468 for 46 in *Other*. Table summarizes total number of publications (TNP), as well as percentages (%) and ranking (RANK) for total publications included in the study and within each taxonomy group. A new index Dcos -*Deciphering Citations Organized by Subject*- to quantify individuals’, research institutes’ or countries’ scientific research output in a particular field is proposed (right side of the table). A detailed explanation of the Dcos index is provided in the corresponding section.

		TOTAL PUBLICATIONS			PUBLICATIONS IN *VIRUS*			PUBLICATIONS IN *BACTERIA*			PUBLICATIONS IN *OTHER*			Dcos INDEX PIG’S INFECTIOUS AGENTS			
		TNP	%	RANK	TNP	%	RANK	TNP	%	RANK	TNP	%	RANK	TOTAL	VIRUS	BACTERIA	OTHER
**USA**	**NA**	915	31.8	1st	425	33.7	1st	338	29.4	1st	152	32.5	1st	915(108)	425(45)	338(34)	152(29)
**GBR**	**EU**	331	11.5	2nd	164	13.0	2nd	140	12.2	2nd	27	5.7	4th	331(83)	164(35)	140(33)	27(15)
**DEU**	**EU**	182	6.3	3rd	82	6.5	4th	65	5.7	5th	35	7.5	3rd	182(58)	82(19)	65(22)	35(17)
**DNK**	**EU**	171	5.9	4th	20	1.6	13th	106	9.2	3rd	45	9.6	2nd	171(50)	20(8)	106(27)	45(15)
**CAN**	**NA**	161	5.6	5th	57	4.5	6th	91	7.9	4th	13	2.8	7th	161(52)	57(22)	91(23)	13(7)
**NLD**	**EU**	139	4.8	6th	68	5.4	5th	60	5.2	6th	11	2.3	10th	139(38)	68(14)	60(19)	11(5)
**ESP**	**EU**	111	3.9	7th	83	6.6	3rd	17	1.5	14th	11	2.3	9th	111(36)	83(18)	17(13)	11(5)
**AUS**	**OCE**	110	3.8	8th	34	2.7	10th	53	4.6	7th	23	4.9	5th	110(42)	34(14)	53(18)	23(10)
**JPN**	**ASIA**	105	3.6	9th	45	3.6	7th	50	4.3	8th	10	2.1	12th	105(51)	45(21)	50(22)	10(8)
**CHN**	**ASIA**	75	2.6	10th	39	3.1	8th	19	1.6	13th	17	3.6	6th	75(36)	39(16)	19(12)	17(8)

When all publications about the 137 studied infectious agents (n = 2,878) were taken into account, Europe coped 45.6% of the papers, whereas North-America hold 38.2%, Asia 10.3% and Oceania 4.6%. South-America and Africa together contributed with less than 1.5% (Fig 4). For each taxonomic group, the observed proportions for every continent were similar to the global percentage reported for that continent, excepting South-America and Africa, where about 50% of the total publications belonged to the group *Other*.

Going down into detail, Table 3 shows the 10 countries with the highest number of publications included in the H-indices. Out of 2,878 publications, USA contributed with 915 (31.8%), having a similar weight in all the taxonomies. The second country with the highest contribution was GBR (331; 11.5%), mostly distributed in *Virus* and *Bacteria* -13.0 and 12.2% respectively- and only 5.7% in *Other*. As seen in Table 3, contributions of countries usually depended on the taxonomy.

Overall, it is important to remark that only three countries contributed with about 50% of the total publications for each taxonomic group. Thus, for all infectious agents USA, GBR and DEU sum up 49.6% of the total; for *Virus* USA, GBR and ESP sum up 53.3% of the publications; a 50.8% of the publications in *Bacteria* corresponded to USA, GBR and DNK; finally, USA, DNK and DEU sum up 49.6% of the publications in *Other*. When rankings were done within a taxonomic group, some countries that were not listed in the general ranking appeared in the top ten; i.e. Belgium was placed 9^th^ in the ranking of *Virus*, Switzerland and Sweden were placed 9^th^ and 10^th^ respectively, in *Bacteria*, and Mexico was placed 8^th^ in *Other* (data not shown).

### Comparison of H-index with other indices

The ten infectious agents with the highest H-index were compared using bibliometric indicators other than H-index, including M quotient, A-index, G-index, HG-index as well as G/H ratio (Table 4).

**Table 4:** Comparison of H-index with other bibliometric indicators for the ten infectious agents with the highest H-indices. Year: year of the oldest publication included in H-index; M quotient: H-index / years from publication of the oldest paper included in H-index; A-index: average number of citations of papers in the H core [19]; G-index [37,38]; HG-index (HG = sr (HxG) [20]; and G/H ratio [37,38].

Infectious agent	Group	Year	H-index (rank)	M quotient (rank)	A-index (rank)	G-index (rank)	HG-index(rank)	G/H ratio(rank)
***Escherichia coli***	BACTERIA	1963	106 (1)	2.04 (3)	153.0 (4)	152 (1)	126.9 (1)	1.43 (9)
***Porcine reproductive and respiratory syndrome virus* (PRRSV)**	VIRUS	1991	95 (2)	3.96 (2)	172.6 (2)	142 (2)	116.1 (2)	1.50 (7)
***Porcine circovirus type 2* (PCV2)**	VIRUS	1995	85 (3)	4.25 (1)	163.6 (3)	132 (4)	105.9 (3)	1.55 (3)
***Swine influenza virus* (SIV)**	VIRUS	1931	79 (4)	0.94 (10)	202.6 (1)	142 (2)	105.9 (4)	1.80 (1)
***Classical swine fever virus* (CSFV)**	VIRUS	1961	72 (5)	1.33 (5)	120.7 (7)	104 (5)	86.5 (5)	1.44 (8)
***Actinobacillus pleuropneumoniae***	BACTERIA	1964	64 (6)	1.25 (6)	120.9 (6)	98 (6)	79.2 (6)	1.53 (5)
***Streptococcus suis***	BACTERIA	1975	62 (7)	1.55 (4)	74.6 (10)	94 (7)	76.3 (7)	1.52 (6)
***Salmonella* Typhimurium**	BACTERIA	1955	61(8)	1.02 (8)	131.8 (5)	94 (7)	75.7 (8)	1.54 (4)
***Aujeszky's disease virus* (ADV)**	VIRUS	1964	58 (9)	1.13 (7)	86.2 (9)	78 (10)	67.3 (10)	1.34 (10)
***Foot-and-mouth disease virus* (FMDV)**	VIRUS	1960	56 (10)	1.02 (8)	119.7 (8)	90 (9)	71.0 (9)	1.61 (2)

When the publication year of the oldest paper included in the H-index core was taken into account, there were noticeable changes in the ranking, especially for SIV; the divisor was the highest among the top ten pathogens and, therefore, its M quotient was the lowest despite its high H-index (rank for H-index = 4; rank for M quotient = 10). The opposite case was represented by PCV2 and PRRSV, which emerged more recently but reached a high H-index: M quotients 4.25 for PCV2 and 3.96 for PRRSV. Also, *Streptococcus suis* improved its position in the ranking from 8th to 4th, because the oldest paper included in its H-index core is relatively recent (1975).

The prioritization of pathogens using the A-index implied SIV changing its ranking compared to the H-index (from 4th to 1st), since it had the highest average of citations for papers included in its H-index (202.6). The pathogen with the lowest A-index was *Streptococcus suis* (74.6).

The prioritization of pathogens obtained by G-index was very similar to rank by H-index, although in this case, again, SIV clearly improved its figure placing in the second position together with PRRSV and surpassing PCV2.

When HG-index scores were calculated from H and G-indices, positions of pathogens in the ranking were practically the same than using H-index alone. On the contrary, G/H ratio produced very different prioritization.

### Deciphering Citations Organized by Subject: the Dcos-index

The research productivity of an individual may be evaluated using several productivity indices. In many cases, the newly described indices try to overcome H-index limitations; however, none of them reflects the importance that publications from a particular author would have within a specific field. In order to quantify the contribution of an individual, either a given author, an institute or a country, in a specific research area or subject, a new index named Dcos (*Deciphering Citations Organized by Subject*), determined by two ures, is proposed. Dcos index would be defined as the number of publications that a certain author, institute or country (among others) holds within the set of papers included in the H-index of a given area or subject. In order to illustrate it, Dcos per countries was measured for swine infectious agents as shown in Table 3. For instance, USA research contribution in viruses affecting pigs would be Dcos = 425(45); meaning that USA contributed with 425 papers in H-index scores of 45 different virus. Thus, Dcos reflects in just two numbers that USA was a great contributor for quantity and also for a large number of viruses. This fact becomes especially clear if we consider that the H-index for 52 virus includes 1,262 papers. More details, examples and potential applications of Dcos index are discussed below.

## Discussion

The use of H-index was originally intended by Hirsch [4] to quantify the output of scientific research for an individual. However, it has been demonstrated that H-index scores may also be useful to rank interest in different pathogens complementing qualitative and other quantitative criteria [14,15,16,39]. However, the method has significant weaknesses and should be accurately applied and interpreted.

### Search accuracy was one of the main cornerstones when analyzing H-index results

One of the tasks with a crucial impact in the final results is to decide which infectious agents and search terms will be included in the analysis. As previous works recognized, to refine the search terms as well as the output lists is very much needed [14,16]. In the present study, a number of searches generated biased lists, introducing several errors. The main error source was the definition of the search terms, because some pathogen names have changed since their discovery, some of them repeatedly. Synonymy for a particular pathogen/disease is very common, especially when a disease emerges and the causal agent remains unknown; for instance, PRRSV and PRRS were known by several names in the past before a unified nomenclature was adopted [21,40]. Therefore, in order to obtain accurate results, search terms should include not only data from NCBI taxonomy or similar, but also from alternative and complementary sources. A second frequent error is the inclusion of numerous papers not related to the topic in the confectioned lists. Several examples for this error are listed below: the search term “PCV2”, included papers exclusively dedicated to other circoviruses like *Chicken anemia virus*. Similarly, the search term “PRRS virus” generated papers related to “pattern recognition receptors” and other pig and non-pig pathogens. Finally, the list of papers for SIV included some papers of *Simian immunodeficiency virus*, even when combined search terms “pig”, “swine” and “porcine” were used. Needless to say that in these cases, the H-index scores could be overestimated. To ensure database accuracy and to minimize the impact of the abovementioned errors or biases, all papers included in the automatically generated list were revised one by one; in some searches, the original generated list was reduced in more than 40%. Obviously, manual revision could be afforded because the amount of terms and organisms to search in the present study was high but limited (2,878 papers overall). In other cases, as in McIntyre and collaborators [16], the number of pathogens analyzed was so high that a strict revision of papers one by one was a Sisyphean task.

### H-index scores were heavily influenced by taxonomic, geographical and temporal features

The number of organisms in each taxonomic group was quite similar: 52 in *Virus*, 39 in *Bacteria* and 46 in *Other*. For the purposes of this work, helminthes, protozoa, external parasites and fungi were fused in the group *Other* because were too small separately. As expected, the weight of viruses and bacteria were significantly higher than *Other* in terms of H-index scores. Although there was no statistical difference, the H-index mean of bacteria was higher than that of viruses, since the number of cases with a low H-index was larger in *Virus* than in *Bacteria*. Independently of the general importance of each infectious agent, the reasons why a high proportion of viruses had a low H-index could be related with the year of detection and/or the distribution. Most emerging organisms were viruses, and some of them emerged in a particular area, country or even in a more limited region within a country, as the *Bungowannah virus* (New South Wales, Australia, 2003) or the *Menangle virus* (New South Wales and Queensland, Australia, 1997), and did not disperse to other regions of the world [21,23,29,41,42]. These restrictedly distributed agents attract few economic resources leading to low H-index scores. Moreover, a recently emerged or re-emerged infectious agent, even having great interest for the scientific community and generating great concern to the pig industry, may not have a high H-index score since some time to accumulate large number of citations is needed. An example would be *Porcine epidemic diarrhea virus* (PEDV). The virus has caused and is still causing severe outbreaks mainly in America and Asia [43]. PEDV was identified as the etiological agent of diarrhea in 1978 [44]; for the following three decades, the infection caused mild outbreaks in Europe and severe outbreaks in Asia only [43,45]. At that time, neither researches nor pig industry, at least not from developed countries, considered PEDV as an important research issue. However, severe large-scale outbreaks related to new PEDV strains were reported in Asia since 2010 and later in the USA and other American countries from 2013 [45]. In Europe, with the single exception of severe outbreaks in Ukraine, outbreaks are milder because PEDV isolates seems to be genetically different compared with those obtained from severe outbreaks in Asia and USA [43]. Nowadays, the international scientific community is doing a great effort to improve the knowledge about highly pathogenic PEDV strains, but some time will be necessary to reflect those efforts in the H-index. Consequently, H-index scores are intrinsically delayed and other measures of the “instant pathogen impact” should be also considered [14].

### The higher the impact on swine health and production worldwide, the higher the H-index scores

Most organisms with the highest 20 H-index scores cause endemic diseases in the largest pig producer countries and show a global distribution. Also, a vast majority were included in databases as DISCONTOOLS, reflecting their importance in swine health. Notable examples are PRRSV and PCV2 that have had a tremendous impact in the last two decades in terms of swine health and economic costs by direct losses and control measures [33,46]. Others, like *E*. *coli*, *S*. Typhimurium or SIV, have also zoonotic potential. In addition, the first two pathogens may have a high H-index because they often display antibiotic resistance. It is noteworthy that searches were done at the species level, and there were no differentiation between non-virulent, mild virulent or highly virulent intraspecific strains or variants, such in the case of *E*. *coli*, which is the causal agent of different significant diseases and lesions in pigs [21]. On the contrary, other pathogens like CSFV, *African swine fever virus* (ASFV) or FMDV appeared in the top 20 because they have a remarkable and inarguable impact in swine health and pig production worldwide. Not surprisingly, the most important pig producer countries in the first world are free from those pathogens [47]. In order to ensure this pathogen-free status, these countries apply strict border controls to limit free-movement of animals and semen, while continuous research efforts are devoted to obtain better vaccines and to control the dissemination of pathogens. Finally, and somehow surprisingly, *Pig endogenous retrovirus* (PERV) was included in the top 20, despite its importance in swine healthy is almost negligible. However, it is a problematic issue in xenotransplantation [48]. Focusing on the group *Other*, a salient feature is the relatively low importance of helminthes, pathogenic fungi, protozoa and external parasites in swine health and pig production, or at least as an issue of scientific interest, as they are. easily controlled by anti-parasitic and anti-fungal compounds. They are frequently found in poor and developing countries, where hygienic conditions in pig farms generally differ from those from developed countries, which concentrate most investments in swine research. To sum up, to the authors’ opinion, almost all pathogens noticed as important by scientists in swine veterinary medicine because of their direct impact to swine health, pig production, or by their zoonotic potential were listed in the top 20 highest H-index scores.

### Similarities and differences between H-index scores: comparison with other published studies

Most pathogens analyzed in the present study were already analyzed by McIntyre and collaborators [16]. When H-index scores were compared, several differences can be observed. Obviously, one of the main causes is that the present study has been undertaken three years later (January 2012 *vs*. March 2015). During these three years, H-index scores may be higher, as they usually increase with time. For instance, this could explain the increase observed in *Actinobacillus pleuropneumoniae* (from 59 in 2012 to 64 in 2015). However, in particular cases, the H-index experienced a marked decrease. The main reason behind this phenomenon could be that the present search was limited to pigs, whereas McIntyre and collaborators (2014) considered domestic, companion and exotic animals used as food sources or pets. Also, it is undeniable that funding differences between only-human and only-animal pathogens are immense. However, other noticeable differences in H-indices obtained between both studies cannot be entirely explained only by these factors. In general, the observed differences pointed out that it is crucial to clearly define the exact search terms and to carefully curate the database afterwards. Interestingly, some pathogens affecting only pigs had a remarkable increase when both studies were compared. Thus, PCV2 and PRRSV improved by 20 and 30 points, respectively, only in three years. Again, the increase may be attributed to the different search dates, but it cannot be discarded that were the result of different search strategies. Similarly, all above discussed may also apply to the prioritization list using H-index described in the EID2 [23]. One weakness of the H-index searches are the presence of not only false positives but also false negatives, caused by not using all search terms for a particular pathogen, or by a non-strict revision of the final list of papers.

### The distribution of H-index scores over time followed several recognizable patterns

Whatever the final H-index score, there is no information regarding the time needed to reach that particular value. When temporal evolution of the H-index is analyzed, crucial features for a particular pathogen could be observed and analyzed, including severe outbreaks, zoonotic outbreaks, emergence in a given country or region, etc. [14]. However, calculations are arduous and extremely time-consuming, because time series of H-index scores must be measured year by year. An alternative and faster method to evaluate the impact of time on H-index is the M quotient. M quotient is simply obtained dividing the H-index score by the years passed since the publication of the oldest paper included in the H-index core. It was proposed to overcome with the temporal H-index dependence, which does not take into account the "age" of the articles. It is especially useful when measuring research impact of young scientists and also for those researchers that *remain silent*, after producing a number of significant articles in the past or they are currently inactive or retired [4]. Likewise, a compound, a researcher or a pathogen/disease could be classified and defined using its M quotient [13,14,49]. In the present study, PCV2 and PRRSV can be clearly defined as *hot topics*, since they reached very high H-index scores in relatively few years, showing the highest M-quotients (>3). The exact limits to define a *hot topic* could be quite subjective [13], but both viruses attract enormous research efforts considering their huge impact in swine health and production. PCV2 reached a high H-index characterized by a constant, sustained increase, despite very efficient PCV2 vaccines appeared worldwide in 2007. Apparently, the description of new variants and the evaluation of different vaccination protocols to improve the protection in piglets could be responsible for the H-index constant increase since then [50]. Regarding PRRSV, its raise to reach the second rank position is easy to understand since: 1) there has been a continuous emergence of new strains that cause severe outbreaks [40,51], 2) commercially available vaccines do not confer full-protection against the infection [52,53], 3) it is considered the major threat for pig production worldwide [21,33], and 4) there are still important gaps in immunology and epidemiology knowledge [53,54]. Quite the opposite, SIV, FMDV and *Salmonella* Typhimurium, could be defined as *sleeping beauties*, since their H-index remained almost inalterable during years, but suffered radical increases within short periods. For SIV, H-index remained almost invariable during decades and had a constant and relatively low annual increase until 2009. Afterwards, in just four years (from 2009 to 2012), its H-index had a relative increase of 25 points. Most probably, this rapid increase was due to the interest of international community related to the global pandemic flu H1N1 [55]. Similarly, FMDV outbreaks in 2001 and 2007 in UK [56] could explain the rapid increase of its H-index related to pig in the last years. Regarding *Salmonella* Typhimurium, its H-index experienced a constant and remarkable increase in the 21^st^ century when the H-index score raised in parallel with the clinical importance of this pathogen [57–59]. Additionally, national control programs applied in some countries might have improved the H-index in this particular case [60,61]. Finally, *Streptococcus suis* improved its rank four positions when H-index and M quotient were compared. This pathogen was firstly described more than 60 years ago [62], but the first paper included in its H-index core was “only” 40 year-old. It seems that H-index for *S*. *suis* has reached a high rate recently because it reflects the increase of human outbreaks in Asia during the last decade [63].

In the present study, H-index scores for nearly all infectious agents with the highest H-indices clearly experienced increases during the 1980´s decade and afterwards, independently of their previous evolution patterns. Radical changes in pig production like artificial insemination, movement of animals, genetics improvement, the continuous industrialization process of farms, changes in management, international trading, etc., have facilitated the spread of pathogens, but also raised research funds for those pathogens affecting pigs. Considering the entire exposed above, plus the improvement of diagnostic tools and reagents currently available for swine research (like PCR, sequencing, ELISA and monoclonal antibodies among others), may explain H-indices increases in swine pathogens experienced during 1980’s and afterwards.

### Mean quartiles did not report differences when the quality of publications was analyzed

The fact that publication impact factor is not considered when measuring H-index is one of the main criticisms of this indicator. In order to consider the impact factor of publications included in the H-index scores, the journal quartile for each published paper accounted in the H-index core was analyzed. However, mean quartile scores among taxonomic groups did not reveal significant differences; neither when the emergence, the zoonotic potential or the OIE declaration was considered (data not shown). A possible explanation may be related to the research area of the journal. A paper about a particular swine pathogen can be submitted to a journal within more than a single research area besides Veterinary Sciences (Virology, Microbiology, etc.). Therefore, similar impact factors may lead to quite different quartiles depending on the research area considered, even more because a given journal can be classified in different quartiles depending on the area of research. Future bibliometric calculations may also analyze the impact factor of the journal and the mean impact factor of the area of research where each paper is published, since it might be a more realistic measure to evaluate the pathogen impact in regards of impact factor of the publications.

### Scientific contributions were highly skewed and show strong geographic patterns

The first author’s affiliation was considered as representative of the geographic origin of the paper authorship. Obviously, this assumption could introduce a bias in the results; however, to the authors’ knowledge, this is the first time that the country of origin of the swine veterinary medicine papers in the H-index cores has been analyzed. The top 20 list of pathogens with the highest H-indices revealed that three countries published more than 50% of the papers included in each H-index. In some cases, like ASFV or PERV, three countries grab more than 90% of the publications. Obviously, research budgets may explain the contribution of each country; besides, the higher the importance of pig industry in a continent or a country, the higher the investment in swine research.

The major contributor to H-index publications in the world was USA, the dominant country in agriculture and food products R&D investment during the last decades [64]. In addition, the weight of pig industry in USA is high, with a census of 6 million of breeding sows in 2014 [65]. But, what can be expected in the future? Certainly, changes in R&D investments should modify the contribution of countries in publications from the H-index core. During the period 2009–2014, a single change in the global R&D investment ranking was reported; China surpassed Japan as 2nd contributor. Besides, the absolute and relative R&D budget has increased only in Southeast Asia, and this trend is expected to continue till the end of the present decade. Actually, if current rates of R&D investment continue for the next years, it is expected that China’s total research budget will exceed that of the USA in about seven years, and the combined of Europe’s 34 countries in only three years [64]. Obviously, this aggressive politics in R&D investment have, and will boost, the impact of China in general research, and specifically in the Veterinary field. According to the SCImago Journal & Country Rank website [66], China has increased by 2500% the number of publications in food animal research area in the period 2000–2014. During the same period, Spain, Germany and USA improved their numbers by 280%, 29% and 38%, respectively. It can be expected that during the next years China’s figures in publications included in the H-index cores of swine pathogens will increase. In addition, it is the major worldwide pig producer with a census of almost 50 million of breeding sows in 2014 [65].

The social-economic situation of a country can also influence its research contribution. As example, R&D budget in Spain is far behind other European countries [67]; however, it is one of the major pig producers in Europe, holding the 2nd largest census in the continent [65,68]. To the authors’ opinion, this panorama could explain why Spain climbed up until position 7th in the general rank of contributors to H-index cores. It is noteworthy that Spain contributed with almost 50% of the papers in the H-index core of ASFV. Currently, Spain is officially recognized as an ASFV-free country, but suffered the disease in the past [69]. That situation, together with the vicinity to Africa could have influenced the scientific and government interest on this particular pathogen, improving the impact of Spain in the ASFV’s H-index. Similarly, most developing countries with a traditional or residual pig production contributed only in the pathogens of their interest. This is especially true for pathogens included in *Other*; several countries in Africa, Asia and South-America appeared only in H-indices of *Other*, where helminthes and, in general, parasites (some of them with a local distribution) are common.

### Some alternatives to H-index and a proposal, the Dcos index: *Deciphering Citations Organized by Subject*

Alternative bibliometrics to H-index have been proposed to overcome H-index weaknesses and limitations [70]. To the authors’ knowledge, this is the first time that several indices have been measured and compared to H-index in order to assess the impact of pathogens. Among them, one of the most known is the G-index. This index tackles one of the H-index limitations, in which the number of citations to each individual publication is ignored [37,38]. As proposed, the G-index gives more weight to those highly cited articles [38] and avoids the weakness of H-index concerning the possibility of “scientists resting on their laurels, since the number of citations received might increase–and consequently their H-index as well -, even if no new papers are published” [3]. G-index takes into account all those citations which exceed the H-index for a given publication and all those citations in papers that do not reach the index. Nevertheless, no significant changes when comparing H-index and G-index rankings were found in the present study. Specifically, only those pathogens that could be involved in zoonotic outbreaks as SIV improved their relative G-index, since some particular papers included in their H-index core accumulated hundreds of citations. In the same way, this phenomenon also influenced the A-index (mean number of citations in the H-index core). Overall, the HG-index rank was almost the same when compared with H-index. On the contrary, the G/H ratio rank, directly influenced by G-index, was quite different. In summary, except for the G/H ratio, that expresses the relation between G and H-indices, and the M quotient, that are related to time to pathogen emergence, the ranking calculated with the H-index is quite similar and representative compared to the other indices measured in the present study.

Several indices have been proposed since Hirsch’s paper coined the H-index [4]. However, none of them captures the significance in terms of the impact that the publication would have within a specific issue. In order to measure it, we propose here a new index named Dcos (*Deciphering Citations Organized by Subject*). Using the H-index core of a pathogen, compound, etc., the Dcos claims to measure the contribution that a given author, institute or country would have in a specific research area or subject. For that, authorship of publications that compose the H-index core should be analyzed. The Dcos index would be determined by two values as stated in the following example: for the total of swine infectious agents, the Research Center for Animal Health in Barcelona (CReSA) had a Dcos = 43(11), where 43 are the number of publications that CReSA’s scientists had in the H-index core of a total of 11 different swine infectious agents. Going down into detail, CReSA’s scientists have 38(7) in swine viruses, 5(4) in swine bacteria and 0(0) for *Other*. The Dcos score for Spain in swine pathogens would be 111(36), which can be further divided into 83(18) for virus, 17(13) for bacteria and 11(5) for *Other*. Bearing this in mind it could be concluded that: 1) in general, CReSA swine research impact could be considered as high in virus, low in bacteria and null in *Other*; 2) this trend is similar to that observed in the whole country; and 3) CReSA’s weight in Spain swine research accounted for about one third for virus and bacteria, but null for *Other*. Dcos can be also detailed to the minimum level, in this case, to a particular pathogen. Using the same example, it can be concluded that research at CReSA, or at least the impact of the research done according to H-indices, seems to be quite concentrated in few pathogens, since its Dcos for swine pathogens was composed by: 15 papers in the H-index core of PCV2, 12 in that of TTSuV, 4 in PRRSV, 2 in HEV, 2 in *Porcine torovirus*, 2 in ADV, 2 in *Mycoplasma hyopneumoniae* and 6 other publications in the rest of H-indices. Also, the index can be applied to individual researchers. In this last case, Dcos would summarize in just two numbers the impact of a particular scientist in his/her field of research. To illustrate it, the Dcos for the authors of the present study was measured: I. Díaz 4(2), M. Cortey 5(2), A. Olvera 5(2) and J. Segalés 35(9). Regarding the first value, it must be reminded that such a number, even being low, represents only those papers within H-index core of a given pathogen, implying papers with the highest number of citations in a particular subject. Obviously, the higher the H-index of a pathogen, the more competitive and the more complicated would be a hit in the final list. Regarding the second value, placed in parentheses, the higher the number, the higher the capacity to conduct a diversified and successful research in different subjects.

## Conclusions

In general, H-index can be used to prioritize the impact of swine pathogens at least for scientific interest. Also, individual evolution of H-index may be useful because reveals important events related to pig production or zoonotic outbreaks. However, the H-index method has important weaknesses, already recognized by Hirsch (2005). When it is applied to rank pathogens, the H-index could be affected by trends in research or funding interest and regional biases, independently of the significance of the agent. Nevertheless, because of its limitations, the H-index method could be used as a preliminary selection in the prioritization of pathogens for investment, or to determine interest on diseases. Moreover, it should be always combined with other methods such as committees of experts and other data to establish a sounder prioritization. In the particular case of swine veterinary medicine, different parameters compared with humans need to be considered to prioritize pathogens. Some of these parameters, which may or may not be reflected in the H-index final score are severity of disease, death rate, economic losses derived from infection or disease, zoonotic potential, spread, persistence and emergence in a given country or in the world. As a final contribution, the Dcos index was proposed, which aims to reflect in just two figures the contribution in a specific research area or subject according to H-index cores of this subject, and if this contribution is focused or diversified.
